# The Digestive System of the Greater Weever (*Trachinus draco* L.) as a Potential Alternative Source of Collagen: A Preliminary Study

**DOI:** 10.3390/ijms27125557

**Published:** 2026-06-19

**Authors:** Nives Kević, Ena Ivić, Jelena Škarica Žikov, Anita Racetin, Marina Rudan Dimlić, Nela Kelam, Ivana Bočina, Ivana Restović

**Affiliations:** 1 Faculty of Science, University of Split, Ruđera Boškovića 33, 21 000 Split, Croatia; nkevic@pmfst.hr (N.K.); ena.ivic3@skole.hr (E.I.); jszikov.biol@pmf.hr (J.Š.Ž.); bocina@pmfst.hr (I.B.); 2 School of Medicine, University of Split, Šoltanska 2A, 21 000 Split, Croatia; amuic@mefst.hr (A.R.); nela.kelam@mefst.hr (N.K.); 3 Mediterranean Institute for Life Sciences, University of Split, Meštrovićevo Šetalište 45, 21 000 Split, Croatia; mrdimlic@medils.unist.hr; 4 Faculty of Humanities and Social Sciences, University of Split, Poljička cesta 35, 21 000 Split, Croatia

**Keywords:** type I collagen, greater weever (*Trachinus draco* L.), gastrointestinal tract, immunofluorescence, hydroxyproline assay, SDS-PAGE, by-product utilisation, histological quantification

## Abstract

This preliminary study characterises type I collagen in the digestive system of the greater weever (*Trachinus draco* L.) by integrating histochemical and biochemical techniques. To the best of our knowledge, this study represents the first baseline mapping of type I collagen within the gastrointestinal tract of this species. Mallory staining and indirect immunofluorescence confirmed collagen presence across the oesophagus, stomach, and intestine. The histochemical quantification of the fluorescent area (100 measurements per organ across 15 fish specimens) showed no significant differences (*p* = 0.1315), indicating a uniform spatial distribution. However, biochemical analysis via hydroxyproline assay and a two-way ANOVA revealed significant differences in collagen content among organs (*p* = 0.0308). The stomach yielded the highest concentration (4.199 µg/mg), significantly exceeding that of the intestine (1.713 µg/mg; Šídák’s post hoc, *p* = 0.0300). This discrepancy suggests that the higher gastric content is due to greater fibre density rather than distribution area. SDS-PAGE and Western blot confirmed protein molecular weights of 100–130 kDa, corresponding to α1 and α2 chains typical of type I collagen. The combination of these histochemical and biochemical methods effectively detects and characterises collagen in fish gastrointestinal by-products. By introducing *T. draco* as a novel subject in this context, these findings provide essential baseline anatomical and histological data and offer a clear scientific justification for the biotechnological valorisation of unutilised commercial fishing by-products, fully aligning with sustainable marine circular economy principles.

## 1. Introduction

Collagen is the major structural protein of the extracellular matrix of connective tissue and one of the most abundant proteins in vertebrates. To date, 28 different types of collagen have been identified, all characterised by the ability to form a helix in which three polypeptide α-chains are tightly wrapped around each other and contain a repeating amino acid sequence (Gly-X-Y)_n_, with the X and Y positions commonly occupied by proline and hydroxyproline [1,2].

The most widespread and thoroughly analysed types of collagen are type I (present in nearly all tissues and organs), type II, and type III [3], which are commonly used in regenerative medicine, tissue engineering, and the pharmaceutical industry as drug effect-prolonging molecules. Types I, II, and III collagen, especially type I, are also used as biomaterials in medicine and cosmetology. Type I collagen accounts for more than 90% of the collagen in the human body and represents over 70% of the entire collagen family [3]. It is mainly found in connective tissues such as joints, cartilage, bone, sclera, ligaments, tendons, intervertebral discs, corneas, the adventitia layer of blood vessels, skin, and the organs of the gastrointestinal and genitourinary tracts [4,5,6]. Type I collagen consists of three polypeptide chains, two identical [α1(I)]_2_ chains and one [α2(I)] chain, each with approximately 1000 amino acid residues [7]. The hydroxylation of proline residues is a typical post-translational modification of type I collagen and is commonly used as a marker to detect and quantify collagen in tissues [8,9].

Due to its unique properties, such as high biocompatibility, biodegradability, and low immunogenicity, collagen has been used in various industries as a primary biomaterial. To meet market demand, terrestrial mammals, primarily pigs and cattle, have long been used as the primary sources of collagen. However, the pathological risks and religious constraints associated with these sources have prompted researchers to seek alternative sources of collagen [10,11,12]. Marine organisms have proven to be significant sources of collagen, as well as many other biologically active molecules [13]. Fish (Pisces) are rich in type I collagen [14], which occurs as fibres that are essential components of connective tissue [1]. Parts of the fish, such as the skin, scales, and internal organs, which become waste during fish processing, are rich sources of collagen. This collagen has demonstrated particular advantages over mammalian collagen molecules [3,15]. Although current research mainly focuses on isolating and characterising collagen from fish skin, the fish digestive system remains underexplored as a potential collagen source [16,17,18]. Collagen is a highly valuable family of proteins used as a biomaterial for nutritional, cosmetic, pharmaceutical, and biomedical applications [3,5,19]. Collagen can be extracted from various sources, and recently, collagen derived from marine organisms has attracted significant attention due to its remarkable properties [12]. Fish processing industries produce large amounts of waste annually, which could be converted into valuable molecules such as type I collagen, an abundant protein found in fish. Thus, the negative environmental impacts of fish by-products could also be reduced [3,11,14].

The physicochemical properties of fish collagen closely resemble those of mammalian collagen, with additional advantages such as abundant availability from aquaculture and by-products, easier extraction, lower zoonotic risk, and enhanced bio-functional properties including low viscosity, non-toxicity, good metabolic compatibility, biodegradability, minimal inflammatory response, and fewer cultural or religious constraints. Despite these benefits, fish-derived collagen has notable limitations, such as low thermal stability, inferior mechanical strength, and rapid degradation rates compared to mammalian collagen [3,20]. Processing fish before sale generates substantial amounts of solid by-products, with more than half the weight of fresh fish becoming by-products of the fish industry [21,22]. Additionally, fish processing results in significant economic losses, with approximately one-third of the fish’s total weight discarded as waste during processing operations [23]. Globally, significant efforts are being made to implement sustainable strategies to prevent food waste and environmental contamination [3]. The valorisation of fish processing by-products would not only mitigate disposal costs but also foster a circular bioeconomy, creating strategic opportunities for the extraction and repurposing of various bioactive substances [24,25], including collagen, which can be utilised in various industries such as the food industry and food packaging, nutraceuticals, cosmetics, regenerative medicine, tissue engineering, and wound healing [3].

The greater weever, *Trachinus draco* (Linnaeus 1758), is a marine fish from the Trachinidae family, best known for its venomous spines on the first dorsal fin and operculum [26]. It is widespread in the Mediterranean and the Black Sea, as well as in the eastern Atlantic, where it can be found from the Norwegian coastline to the islands of Madeira and Morocco [27]. The greater weever is biologically important for its ecological role in the demersal ecosystem and as a food source in some regions. It has a special commercial value, most often caught as by-catch and sold at local fish markets [28,29]. The greater weever is widely distributed in the Adriatic Sea, mostly in channel areas, living on sandy and muddy substrates along the upper slope of the continental shelf at depths ranging from a few metres up to 100 metres. This suggests that warmer sea temperatures may be preferred by the greater weever for feeding [28], making this fish species suitable for type I collagen isolation. As stated by Rajabimashhadi et al. (2023) [3], the low denaturation temperature of fish collagen is due to its evolutionary adaptation to aquatic habitats and is one of the few disadvantages of using collagen from fish by-products. The only possible approach is to select the fish species carefully, choosing one that inhabits warmer environments and therefore has collagen with a physiologically higher denaturation temperature compared to species from colder aquatic environments. Isolated collagen with a physiologically higher denaturation temperature can be used in various industrial fields, particularly in certain clinical applications [30].

By burying itself in the sea floor, exposing only its eyes and the tip of its dorsal fin, this predator ambushes its prey [26,31]. The venom of the greater weever can cause swelling, fever, dizziness, difficulty breathing, and severe pain in the victim, which is disproportionate to the size of the small envenomation puncture wounds [26,32]. Scientific studies on the greater weever have primarily focused on toxin properties (dracotoxin) and envenomation [33,34,35,36]. Fish venoms can cause cardiovascular, neuromuscular, oedematous and cytolytic symptoms, with cardiovascular activity noted as the main effect after envenomation [32,37]. The venoms consist of a mixture of toxins, enzymes, bioactive peptides, and non-protein components such as serotonin, norepinephrine, and acetylcholine [38]. Kula et al. (2020) isolated new bioactive peptides from the myofibrillar proteins of the greater weever [39], suggesting that this species has significant potential as a source of biologically important molecules for future biomedical applications, as these peptide molecules have shown ACE inhibitory, DPP4 inhibitory, antioxidant, and metal chelation activities. It is considered that the greater weever’s biological potential has not been fully explored, and therefore, its role as a suitable source of collagen molecule has been examined.

As scientific and industrial interest in marine collagen is currently increasing, this study aimed to characterise the presence and distribution of type I collagen within the gastrointestinal tract (the oesophagus, stomach, and intestine) by integrating histochemical methods, the indirect immunofluorescence technique, hydroxyproline assay, SDS-page electrophoresis and the WB technique. By evaluating these tissue-specific structural characteristics for the first time in this species, this preliminary assessment explores the molecular potential of commonly underutilised commercial fishery waste. Successfully identifying collagen type I in the digestive system of *T. draco* could indicate that this material, which would otherwise be discarded, has the potential to serve as a valuable alternative resource for various industrial purposes, fully aligning with sustainable marine circular economy principles.

## 2. Results and Discussion

### 2.1. The Histochemical Staining of the Digestive System in the Greater Weever

The digestive system of the fish, as in other vertebrates, consists of the digestive tract and associated glands [40]. The digestive tract includes the oral cavity, pharynx, oesophagus, stomach, intestine, and anal opening. Histologically, from the cranial end of the oesophagus to the caudal end of the rectum, the wall of the fish digestive tract is generally described as being composed of four layers [41]. The basic structure of the wall is relatively consistent throughout the entire length of the tract [42,43]. Starting from the lumen, the wall consists of four distinct layers: the mucosa (tunica mucosa), submucosa (tunica submucosa), muscle layer (tunica muscularis) and outer layer (tunica serosa or tunica adventitia) [44,45]. However, structural variations exist among specific regions; in many teleost species, the intestinal wall lacks a distinct muscularis mucosae, leading to the fusion of the lamina propria and submucosa into a single lamina propria–submucosa layer, which functionally presents a three-layered organisation [44].

**Figure 1:**
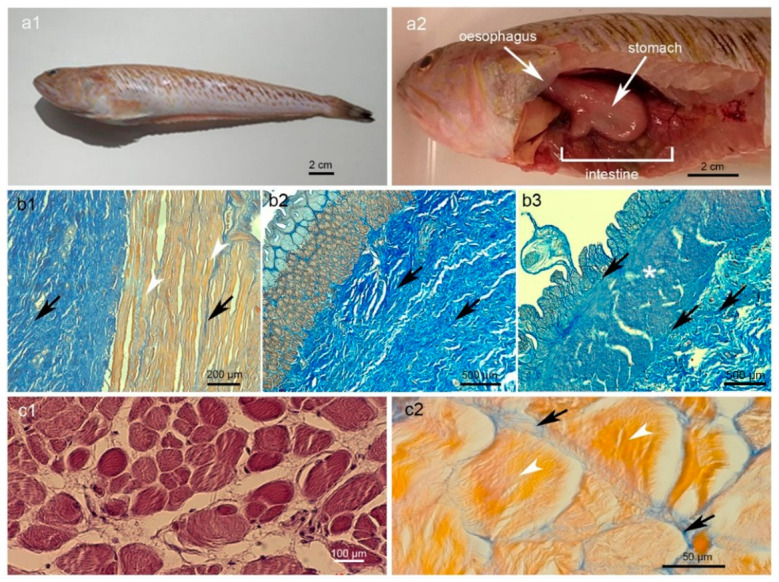
Greater weever *T. draco* (**a1**). Anatomical section of digestive tract of greater weever (**a2**). Cross-section through submucosa and muscle layer of oesophageal wall, scale bar 200 μm (**b1**). Cross-section through mucosa and submucosa layers of stomach wall, scale bar 500 μm (**b2**). Cross-section through submucosa and muscle layer of stomach wall, scale bar 500 μm (**b3**). Skeletal muscles of oesophagus, H&E, scale bar 400 μm (**c1**). Skeletal muscles of oesophagus, Mallory, scale bar 100 μm (**c2**). (**b1**–**b3**,**c2**) Mallory staining. (**c1**) Haematoxylin–eosin staining. Collagen fibres (arrow); skeletal muscle fibres (white arrowhead); smooth muscles (asterisk).

A cross-section through the oesophageal wall in the greater weever shows these four layers as described. The connective fibres of the oesophageal mucosa and submucosa were stained blue with the Mallory technique, indicating the presence of collagen (Figure 1b1), while the orange colouring in the muscle layer indicated the presence of skeletal muscle tissue (Figure 1b1,c2). In a study by Bralić (2010), the muscular layer of the oesophagus in the greater weever was found to consist of two layers of skeletal muscle: an inner longitudinal layer situated on the submucosa and an outer circular layer, visible as fibres displaying striations [46]. Cross-striations, characteristic of skeletal muscle, are clearly seen in H&E micrographs of the tunica muscularis of the greater weever (Figure 1c1). Connective tissue is present between the muscle layers [46].

The collagen fibres in the connective tissue of the gastric mucosa and submucosa were stained blue with Mallory staining, indicating the presence of collagen (Figure 1b2). The gastric muscle layer was stained purple, indicating the presence of smooth muscle tissue in the stomach of the greater weever (Figure 1b3), in contrast to the muscle layer in the fish oesophagus (Figure 1c1,c2). Mallory staining highlights collagen fibres in blue within histological specimens, serving as a key method for visualising connective tissue in medical diagnostics and research, especially in histopathology for the detection of amyloid deposits in tissues and in connective tissue research. Consequently, this technique is essential for studying the structure and pathology of various connective tissues [47].

However, as previously noted in some species, such as the greater weever, the intestinal wall exhibits only three distinct layers (mucosa, muscularis, and serosa) compared to the layers found in the oesophageal and gastric walls [46,48].

### 2.2. Immunolabelling of Type I Collagen Protein in Digestive System of Greater Weever

**Figure 2:**
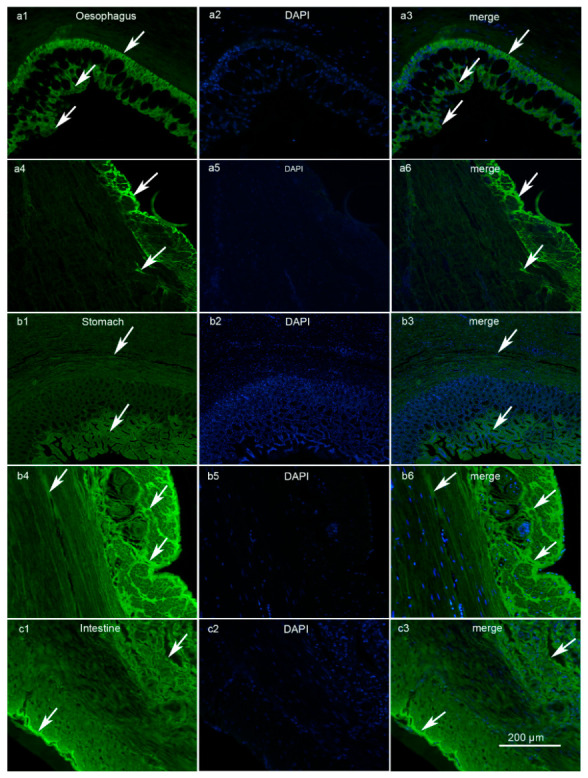
Expression of type I collagen (arrows): (**a1**–**a3**) mucosa and submucosa layers of oesophagus; (**a4**–**a6**) muscle and outer layer of oesophagus; (**b1**–**b3**) mucosa and submucosa layers of stomach; (**b4**–**b6**) muscle and outer layer of stomach; (**c1**–**c3**) intestine wall. Scale bar 200 μm.

When comparing the distribution of type I collagen in different regions of the greater weever digestive system, it was observed that collagen is present in all investigated regions. These findings strongly support the proposition that the digestive system, as a primary fish processing by-product, serves as a highly valuable and abundant source of collagen (Figure 2). The immunolocalisation of type I collagen was detected in all layers of the oesophagus, stomach, and intestine of the greater weever, except for the lamina epithelialis in the mucosal layer of the intestinal wall (Figure 2).

Valid immunolocalisations of type I collagen were observed throughout the mucosa and submucosa of the oesophagus (Figure 2a1–a3). Significantly strong signals were detected in the epithelial cells, on the surface and beneath the goblet cells in the mucosal layer, in the lamina propria of the mucosa, and in the connective tissue of the submucosa (Figure 2a1–a3). Very strong immunofluorescence was also observed in the muscle layer and the outer layer (adventitia) of the oesophagus. The observed strong signals clearly indicated the presence of type I collagen at the border of the submucosa and muscle layer, in the connective sheaths around the muscles, and in the outer muscle layer of the oesophagus (Figure 2a4–a6).

Additionally, a moderate immunolocalisation of type I collagen was observed throughout the stomach wall and in the epithelium of the mucosa and submucosa layer (Figure 2b1–b3). However, strong collagen expression was detected in the connective tissue of the outer layer, the serosa, while moderate expression was found in the connective tissue of the muscle layer (Figure 2b4–b6).

The lamina propria of the intestinal mucosa, the connective tissue of the muscle layer, and the outer serosa all exhibited a moderate intensity of type I collagen. However, no collagen signal was detected in the mucosal epithelium (Figure 2c1–c3). This may be strongly correlated to the findings in human intestinal epithelial cells, which have been shown to synthesise type VI collagen. Collagen VI was localised throughout the lamina propria and the surrounding blood vessels and appeared to be concentrated in regions at the epithelial/stromal interface [49].

### 2.3. Statistical Analysis of Type I Collagen Expression in Fish Gastrointestinal Organs

**Figure 3:**
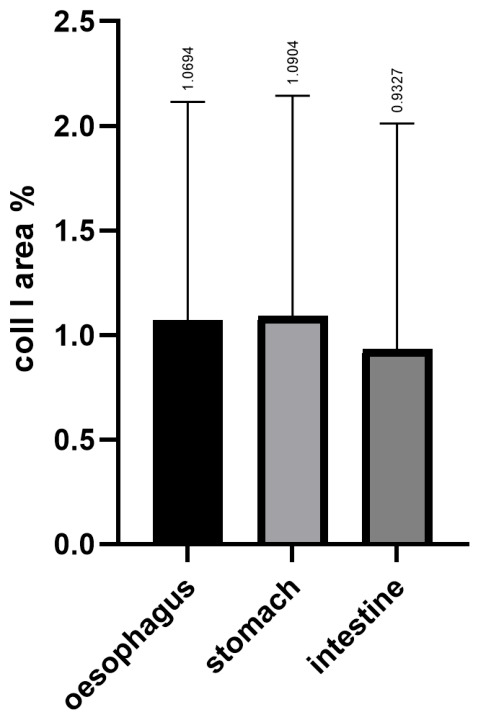
A statistical analysis of type I collagen expression across three different regions of the greater weever’s digestive system. Expression was quantified by measuring the fluorescence area percentage. The data are presented as the mean ± standard deviation (M ± SD).

Significant percentage areas of type I collagen expression were detected in the oesophagus, stomach, and intestine, confirming that all three regions of the greater weever digestive system have potential as alternative sources of type I collagen (Figure 3). Among the three gastrointestinal regions, the intestine had the lowest percentage area of collagen (Figure 3). This is probably due to the histological structure of the intestinal wall, which frequently contains three layers rather than four—often characterised by the lack of a distinct muscularis mucosa or the fusion into a lamina propria–submucosa—as typically found in the more complex walls of the oesophageal and gastric regions. This anatomical simplification directly correlates with the lower relative abundance of connective tissue, and consequently type I collagen, in the intestinal region compared to the more muscular and structured oesophageal and gastric zones.

Statistical analysis (Table 1) confirmed that the data followed a normal distribution (Shapiro–Wilk test, *p* > 0.05). While the percentage area of collagen expression was numerically the lowest in the intestine, no statistically significant difference was found between the three regions (one-way ANOVA, *p* = 0.1315). However, a noticeable numerical trend was observed, with the intestine exhibiting an approximately 13% lower mean collagen area compared to the stomach and oesophagus (0.932 vs. 1.090, respectively). This lack of statistical significance despite the numerical variance suggests that the 2D histochemical analysis might be underpowered to detect subtle spatial differences, and caution should be exercised before assuming identical collagen distribution across all regions. Interestingly, the observed heteroscedasticity (Brown–Forsythe, *p* = 0.0051) suggests that while the mean expression is similar, the spatial consistency of collagen distribution varies significantly between the organs. Furthermore, the statistical analysis revealed high standard deviations relative to the mean values across all organs (Table 1). This wide data dispersion represents a methodological limitation inherent to high-resolution histochemical quantification in complex tubular organs. Because the gastrointestinal wall consists of alternating micro-environments, ranging from collagen-dense submucosal layers to epithelial structures and mucosal folds with minimal connective tissue, individual microscopic fields of view naturally capture highly contrasting fluorescence areas. While this high inter-sample variability reflects the true, non-homogenous spatial distribution of collagen within the tissue matrix, conducting a high number of measurements (100 per organ) effectively mitigated this limitation, ensuring a precise and reliable estimation of the overall mean area fraction. This extensive sampling protocol allowed for a precise estimation of the collagen area fraction, ensuring that the captured numerical trends reflect genuine tissue architecture despite the inherent biological variability. Additionally, as shown in Table 1, while the SD is wide due to the tissue architecture, the Standard Error of the Mean (SEM) remains low and tightly controlled (~0.10), confirming that our large sample size provided an accurate and statistically sound estimation of the true mean collagen area fraction.

### 2.4. Statistical Analysis of Collagen Quantification—Hydroxyproline Assay in Fish Gastrointestinal Organs

**Figure 4:**
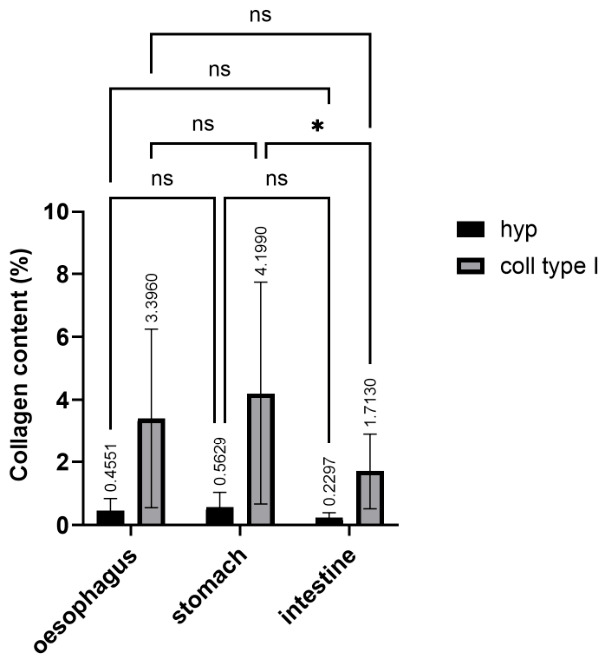
A comparative statistical analysis of hydroxyproline and collagen content within three distinct organs of the greater weever digestive tract. The data are presented as the mean ± standard deviation (M ± SD). Statistically significant differences (*p* < 0.05) determined by Šídák’s post hoc test are indicated by asterisks (*), while “ns” denotes non-significant differences (*p* > 0.05).

We analysed the amount of collagen in fish samples (Table 2, Figure 4). Hydroxyproline (Hyp) was used as an indirect marker of collagen quantification, as its concentration is directly proportional to the collagen content in the tissue. The initial mass of the tissue sample was 10 mg, and the total volume of the reaction mixture was 200 µL. A standard curve was constructed using known hydroxyproline concentrations (0–1.0 µg/well), and the results showed high linearity (R^2^ ≈ 0.999). Based on the obtained absorbances, the concentrations of hydroxyproline per milligram of sample were calculated and then converted to collagen using the factor 7.46 (1 µg of hydroxyproline = 7.46 µg of collagen) [50]. The results showed that the stomach samples had the highest average collagen content (4.199 µg/mg), followed by the fish oesophagus samples with 3.396 µg/mg (Table 2). In comparison, the lowest values (1.713 µg/mg) were recorded in the intestine (Table 2). Additional verification of the method’s accuracy was performed using spiked controls, in which known amounts of hydroxyproline were added to the samples.

**Figure 5:**
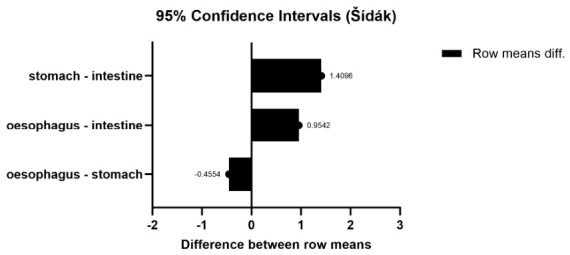
An estimation plot of the differences between row means (organs) with 95% Šídák’s confidence intervals. The values indicated at the end of the bars represent the mean differences. A comparison is statistically significant when the 95% CI does not include zero (line of no difference), as seen in the stomach–intestine pair. Comprehensive ANOVA parameters are detailed in Table 3.

Statistical analysis performed via a two-way repeated measures ANOVA confirmed significant differences among the studied digestive organs (*p* = 0.0308) and between the analysed parameters (*p* = 0.0004), as illustrated in Figure 4 and Figure 5 and detailed in Table 3.

Differences across the organs, as well as the relationships between morphometric (% area) and biochemical (hydroxyproline) datasets, were comprehensively evaluated using the global two-way repeated measures ANOVA. To further investigate the differences between organs, Šídák’s multiple comparisons test was performed on the row means (Figure 5, Table 3).

The biochemical quantification of collagen content revealed a clear distinction between the organs, where the comparison between the stomach and intestine was the only one in which the 95% confidence interval (0.1099 to 2.7096) did not cross zero, confirming a statistically significant higher content in the stomach with a mean difference of 2.486% (*p* = 0.0300) (Figure 5, Table 3). While the 95% confidence interval for this comparison is relatively wide, reflecting the inherent biological variability in and varying hydration states of the gastrointestinal tissues among wild specimens, the distinct separation from zero strongly supports the robustness of the statistical significance. Conversely, the analysis of the fluorescent area percentage showed no significant differences across the same regions (*p* = 0.1315, *p* > 0.05). The notable discrepancy between the uniform 2D spatial distribution (histochemical area) and the significantly different chemical mass (biochemical hydroxyproline assay) highlights the structural complexity of the fish gastrointestinal matrix. While immunofluorescence highlights the specific microanatomical localisation of type I collagen within the tissue layers, the hydroxyproline assay provides a holistic quantification of total collagen. These two approaches should be viewed as complementary rather than directly proportional; histochemical quantification via image analysis calculates the percentage of a two-dimensional area, which inherently fails to account for three-dimensional density, fibre thickness, or the molecular cross-linking of the proteins [51]. Biologically, the stomach is subjected to intense mechanical forces during physical digestion and food storage, requiring a highly resilient extracellular matrix [52]. Our biochemical findings (4.199 µg/mg in the stomach vs. 1.713 µg/mg in the intestine; *p* = 0.0300) suggest that the gastric wall achieves this mechanical strength through a significantly higher density of tightly packed collagen fibres and potentially greater intermolecular cross-linking, rather than by simply expanding the total surface area of collagen distribution [52,53]. This architectural divergence demonstrates that biochemical content cannot be accurately predicted by 2D histological area alone, highlighting the stomach as the primary candidate among the investigated by-products for mass collagen extraction.

The quantification of collagen in specific tissues is often carried out through hydroxyproline analysis [50,54]. Collagen content has been measured in the skin of various fish species, including cartilaginous fish such as the small-spotted catshark (*Scyliorhinus canicula*) [55] and blue shark (*Prionace glauca*) [56], and in bony fishes such as yellowfin tuna (*Thunnus albacares*) and swordfish (*Xiphias gladius*) [55], Japanese sea bass (*Lateolabrax japonicus*) and Nile tilapia (*Oreochromis niloticus*) [57]. In experimental samples of catfish *Silurus triostegus,* collagen content in the muscle, head, fins, bone, and skin ranged from 15 to 24% [11]. This is similar to that of *Nile perch* skin collagen, containing 20 to 22% protein [58], as well as that of leatherjacket fish [59]. Collagen isolated from the scales of different fish species has also been reported [60]. Nishimoto et al. (2009) [61] report that the fish digestive tract, which often ends up as waste in large quantities, could be a potential source of collagen. Type I collagen is the predominant type found in the digestive tract; however, it differs from skin collagen regarding the degree of hydroxylation of its prolyl and lysyl residues [61]. Currently, most research focuses on the isolation and characterisation of collagen from fish skin, while the digestive system as a potential source of collagen remains an almost completely unexplored topic.

To our knowledge, there are no data in the available literature on the content and quantity of type I collagen in the gastrointestinal system of the greater weever, making this the first study to establish these tissue-specific structural characteristics for this species. Nishimoto et al. (2009) [61] reported that large quantities of the digestive tract, stomach, intestine, and adjacent tissues are generated by the fishery industry, and most of these are low-value by-products. It has been demonstrated that the collagen in the digestive tract of the Japanese amberjack (*Seriola quinqueradiata*), a predatory and highly valued culinary fish, differs from the skin collagen in terms of solubility, post-translational modification and molecular species composition. The main collagen in the digestive tract is identified as type I collagen, in which the degrees of hydroxylation of prolyl and lysyl residues are higher than in skin collagen, suggesting the greater stability of the collagen from the digestive tract [61].

Our preliminary research confirmed the presence of type I collagen in the gastrointestinal system of the greater weever, in concentrations ranging from 1.7 to 4.2%. Referring to Nishimoto’s study [61], which demonstrated that the total collagen content isolated from digestive organs was 2% of wet tissue, we showed that greater weever stomach samples had an average collagen content of approximately twice that level (~4.2%) compared to Nishimoto’s findings. However, it is important to acknowledge that this direct comparison is inherently confounded by differences in experimental methodologies; while Nishimoto [61,62] quantified the final yield of isolated collagen from wet tissue, our study determined the total biochemical collagen content via hydroxyproline assay. Given that extraction procedures rarely achieve 100% efficiency, and that definitions of wet tissue hydration states can vary, these values should be interpreted as a comparative trend rather than an absolute baseline divergence in species-specific collagen abundance. Nevertheless, this distinctive biochemical profile highlights that the greater weever’s gastrointestinal tract holds substantial structural matrix potential.

It has been reported that the fish digestive tract is a potential source of collagen-derived products (gelatine and its enzymatic hydrolysate) when compared with the total collagen content isolated from white (0.3%) and red muscle (0.8%) in Japanese amberjack [62]. Based on the characterisation of collagen from the greater weever’s gastrointestinal system, these processing by-products represent a promising alternative collagen source for various industries, including the biomedical, pharmaceutical, and food sectors.

While most literature identifies skin, bone, scales, and muscle as optimal collagen sources [63], our preliminary results show a total collagen content that is comparable to the minimum yields reported for those tissues while simultaneously approaching the maximum yields reported for notochord-based collagen [3,63]. This comparison is noteworthy because the yield of collagen extraction from animal by-products varies depending on the source, the age and type of animal, the condition of the by-products after processing, and the extraction technique used [3]. Reported collagen extraction yields vary greatly, ranging from as little as 0.05% to nearly 95% [3]. By evaluating these overlooked fishing by-products, our work could provide a valuable foundational framework for assessing their molecular potential. Introducing *T. draco* into this biotechnological conversation may provide a sound scientific justification for the future valorisation of unutilised biomass, potentially aligning with sustainable marine circular economy principles.

### 2.5. SDS-PAGE and Western Blot of Collagen Extracts from Fish Gastrointestinal Organs

Fibrillar collagen, such as type I collagen, consists of three alpha (α) chains: two α1 chains and one α2 chain that self-assemble into a left-handed triple helix structure. The pepsin digestion of collagen removes the telopeptide portion, which is highly immunogenic [64]. In ageing tissues, collagen develops intra- and inter-collagen cross-links between lysine residues in the α-chains due to oxidation. The intra-collagen cross-links can result in two specific structures: a beta (β) chain (cross-links between two α-chains) and a gamma (γ) chain (cross-links between three α-chains). SDS-gel analysis can be used to assess and evaluate collagen quality and production, where a single α-chain has a molecular weight of 100 kDa, and a β-chain has a molecular weight of 200 kDa. The ratio of α-, β-, and γ-chains in a collagen gel analysis depends on the degree of cross-linking in the sample [64].

**Figure 6:**
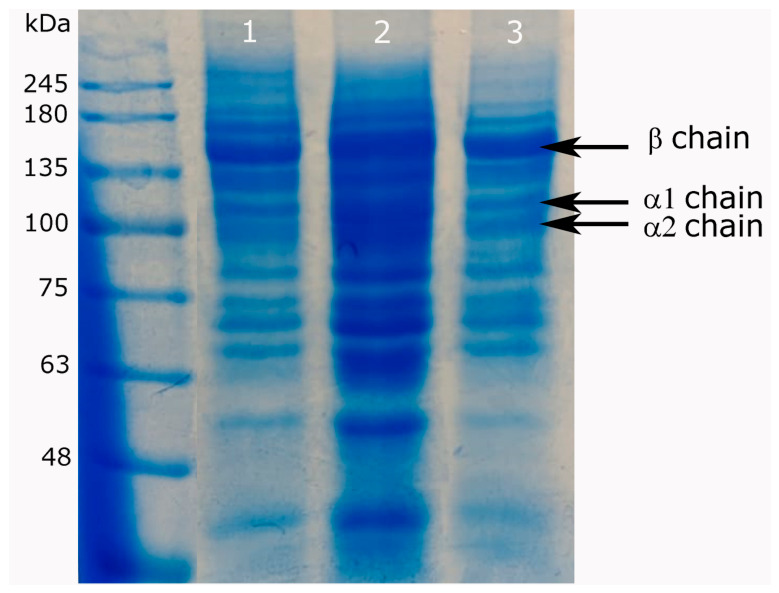
An SDS-PAGE analysis of pepsin-solubilised collagen from the greater weever’s digestive organs (1—oesophagus, 2—stomach and 3—intestine). A molecular weight marker for estimating the molecular weights of the analysed collagen protein bands is shown on the left side of the gel.

**Figure 7:**
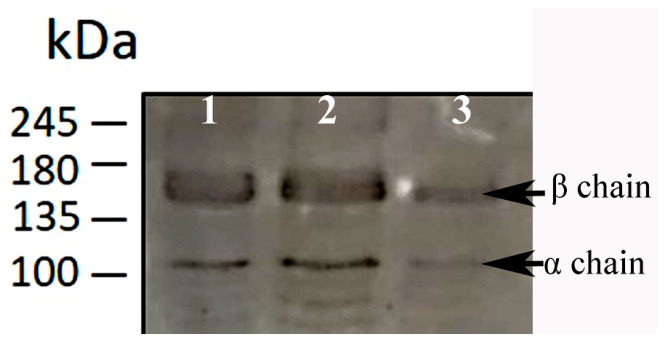
A Western blotting analysis of pepsin-solubilised collagen from the greater weever’s digestive organs (1—oesophagus, 2—stomach and 3—intestine). A molecular weight marker for estimating the molecular weights of the analysed collagen protein bands is shown on the left side of the film.

Figure 6 and Figure 7 show the collagen gel pattern from the greater weever gastrointestinal organs, isolated by SDS-PAGE and Western blotting analysis. The SDS-PAGE analysis of pepsin-extracted collagen (Figure 6) in our study showed an electrophoretic profile characteristic of type I collagen. Two dominant protein bands were observed in the molecular weight range of approximately 100–130 kDa, corresponding to the α1 and α2 chains. The intensity ratio of the bands (~2:1) confirms the presence of type I collagen, which consists of two α1 and one α2 subunit. Highly visible bands of higher molecular weights (~200–250 kDa) were also detected, attributed to β-dimers, while the γ-components were weakly expressed or absent.

Our Western blot results (Figure 7) are the first to report the collagen molecule profile isolated from the gastrointestinal organs of the greater weever, confirming the presence of type I collagen in the digestive tissue extracts. Immunoreactive bands were detected at the same molecular masses as in the SDS-PAGE analysis (Figure 6), confirming the presence of collagen α- and β-chains. The signal was the most pronounced in the area of the α- and β-chains, while a very weak signal was observed in the area of higher molecular mass (γ-component) (Figure 6 and Figure 7).

**Figure 8:**
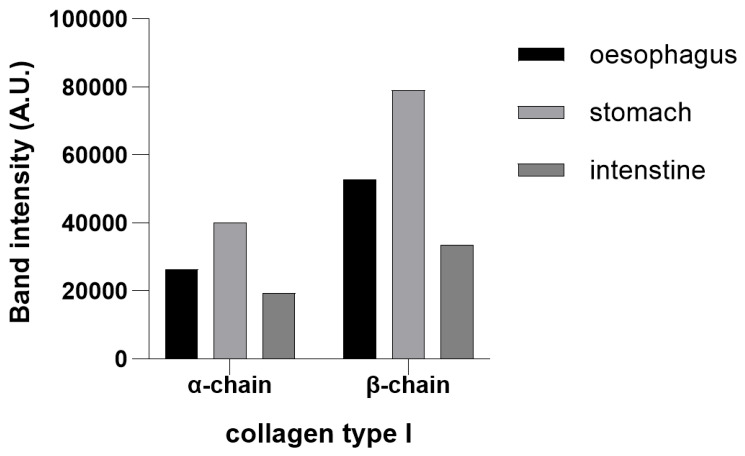
The densitometric quantification of collagen α-chain and β-chain protein bands. Band intensities across the oesophagus, stomach, and intestine of the greater weever were quantified using ImageJ software. Data are presented as net integrated density values (arbitrary units) after background subtraction to reflect relative expression levels among the analysed tissues.

To further evaluate these expression patterns, a densitometric quantification of the Western blot bands was performed (Figure 8). The analysis confirmed that the β-chain exhibited a higher overall net integrated density compared to the α-chain across all investigated tissues. Comparing samples obtained from the oesophagus, stomach, and intestines of the greater weever fish, the densitometric profiles suggest that the collagen signal intensity is the strongest in the oesophagus and particularly in the stomach. Conversely, the intestine showed the weakest signal for both the α- and β-chains using these extraction techniques. The combination of SDS-PAGE and Western blot analysis in our research confirms that the applied extraction method is effective for isolating collagen from the digestive organs of the greater weever. The obtained electrophoretic profile corresponds to a typical collagen protein pattern.

In this study, these tissue-specific variations in collagen levels were monitored through the direct band intensity values presented in Figure 8. To ensure an accurate and reliable comparison of signal intensities across the oesophagus, stomach, and intestine without the use of an internal housekeeping control, equal amounts of total protein were strictly loaded into each lane, and the samples were resolved on a single gel under identical experimental conditions. While this approach provides semi-quantitative insight, the prominent differences in the net integrated density values strongly support a biologically driven gradient in collagen abundance, suggesting the stomach to be the most prominent source of type I collagen among the analysed digestive organs.

Furthermore, our findings align with recent research on collagen extracted from catfish (*Silurus triostegus*) by-products, where SDS-PAGE analysis revealed molecular weights ranging from 97 to 200 kDa, comprising both α- and ß-chains [11]. Additionally, collagen from catfish skin, bone, and fins consisted of two distinct α-chains, a characteristic feature of type I collagen [11]. These observations are consistent with studies by Xu et al. on catfish skin [65], Ben Slimane and Sadok on *Mustelus mustelus* skin [66], Muyonga et al. on Nile perch skin [58], and Skierka and Sudowska on Baltic cod [67], which similarly identified the isolated proteins as type I collagen. Notably, while Nishimoto et al. [61] identified type I collagen from the skin and digestive tract of Japanese amberjack using SDS-PAGE, to our knowledge, this is the first report of a collagen protein profile from the digestive system of the greater weever (*T. draco*) verified by both SDS-PAGE and Western blot analyses.

### 2.6. Limitations of This Study

Although this preliminary study provides valuable and methodologically sound insights into the collagen profile of *T. draco*, certain limitations should be acknowledged. Firstly, the biochemical quantification via hydroxyproline assay was conducted on a sample size of 15 specimens. Wild fish populations inherently exhibit biological variability driven by fluctuating environmental conditions, individual dietary habits, age, sex, and specific physiological maturity stages. Due to the commercial nature of the catches, precise data regarding these individual variables (such as sex and exact maturity stages) were unfortunately not available. To mitigate these factors and ensure the maximum standardisation, all specimens in this study were strictly selected from the same commercial catch cohort and fell within a uniform size and weight range representing morphologically homogenous, mature adults.

Furthermore, it is important to note that the holistic quantitative mapping in this study focused exclusively on the direct biochemical quantification of total collagen content within the tissue matrices and the measurement of the immunoexpressed surface area, rather than performing an experimental protein extraction to isolate collagen in powder form for commercial yield assessment. Consequently, our quantitative data reflect the absolute biological presence of collagen within the intact tissues, which limits direct comparison with studies measuring isolated collagen yields where extraction-related losses may occur. While our statistical analyses (two-way ANOVA) demonstrated sufficient statistical power to detect significant differences in collagen content between the stomach and intestine, caution should be exercised when generalising these absolute quantitative values to broader geographical or multi-seasonal populations of *T. draco*. Furthermore, as a preliminary characterisation focused primarily on spatial distribution and total collagen content, future comprehensive studies incorporating a larger sample size across different life stages and seasonal cycles would be beneficial to fully map the physiological variability in type I collagen in this species.

## 3. Materials and Methods

### 3.1. Experimental Animals and Tissue Collection

Fifteen greater weever (*T. draco*) specimens, freshly caught at the Blitvenica location, Žirje Island, Croatia, in the Adriatic Sea (coordinates 43°33′35.8″ N 15°30′19.78″ E) in September 2024, were used for this study. To minimise the inherent biological variability common in wild fish populations, all specimens were strictly selected from the same commercial catch and represented mature adults. The average length of the greater weever specimens was 27.2 cm, with the average weight being 67.95 g. Digestive system samples from each fish were sectioned at the oesophagus, stomach, and intestine regions and prepared for histochemical, immunofluorescent, and SDS-PAGE/Western blot analyses.

### 3.2. Histochemical Procedure

Immediately after collection, an anatomical section of the digestive system was performed, and sections of the oesophagus, stomach, and intestine were fixed in 10% formalin solution (Figure 1a2). After fixation, tissue samples were dehydrated in an ascending series of ethanol, cleared in xylene, and embedded in paraffin wax (Paraplast Plus, Leica, Deer Park, IL, USA) at 56 °C on a HistoCore Arcadia H (Leica Biosystems, Wetzlar, Germany). Paraffin sections of 6 μm thickness were cut on a HistoCore BIOCUT rotary microtome (Leica Biosystems, Nussloch, Germany) and mounted on glass slides [45,68].

#### 3.2.1. Haematoxylin–Eosin Staining

Haematoxylin–eosin staining was used to present the basic morphology of the tissue samples. The sections were first deparaffinised with xylene and rehydrated with ethanol and water. Haematoxylin (Haematoxylin M, BioGnost, Zagreb, Croatia) and 1% eosin (Eosin Y 1% aqueous, BioGnost, Zagreb, Croatia) staining were then applied for 15 min and 5 min, respectively. The slides were observed using an Olympus BX51 light microscope (Olympus, Tokyo, Japan).

#### 3.2.2. Mallory Staining

The paraffin sections were first deparaffinised through a series of xylenes and brought to distilled water through a downgraded ethanol series. Postfixation with 3% Möller solution (3% potassium bichromate in 4% formalin solution) was applied to the sections for 2 h. After washing in distilled water, the sections were heated for 30 s with 1% acid fuchsin. After rinsing in distilled water, the sections were immersed in 1% phosphomolybdic acid for 15 min to fix the colour and then in aniline blue–orange G solution for 2 min. The nuclei were stained with haematoxylin for 10 min, rinsed in distilled water, and blued in warm tap water for 10 min before acid fuchsin staining was applied. Finally, the sections were washed with distilled water, dehydrated through an ascending series of ethanol, cleared in xylene, and mounted in NeoMount (Merck, Darmstadt, Germany). The slides were observed using a Leica DM3000 LED light microscope (Leica Microsystems, Wetzlar, Germany).

### 3.3. Immunofluorescent Labelling Procedure

After deparaffinisation and dehydration, the sections were heated for 10 min in 0.1 M citrate buffer (pH 6.0) in a water steamer, then cooled to room temperature and placed in a humidity chamber for immunofluorescent labelling [69,70]. Before applying the blocking buffer, the tissue sections were incubated with 0.2% hyaluronidase solution (approximately 500 U/mg) for 15 min at 37 °C, then rinsed with PBS for 5 min. Blocking buffer (ab64226, Abcam, Cambridge, UK) was applied for 20 min to prevent nonspecific staining. Sections were then incubated overnight in a humidity chamber with the primary antibody diluted 1:80 (Table 4). After washing in PBS, secondary antibody Alexa Flour 488 (Table 4) was applied for one hour and washed in PBS again. Nuclei were stained with 4′,6′-diamidino-2-phenylindole (DAPI) for 2 min. After a final wash in PBS, sections were mounted in Aqua-Poly/Mount (Polysciences, Warrington, PA,, Germany). All slides were examined using an Olympus BX51 epifluorescence microscope (Olympus Corporation, Tokyo, Japan) with a Nikon DS-Ri1 digital camera (Nikon Corporation, Tokyo, Japan) [69,70].

Tuna fish skin, a tissue well-documented for its high type I collagen content and cross-reactivity with this fish-specific antibody, was utilised as a positive internal control to confirm effective binding. Conversely, negative controls were performed by omitting the primary antibody and incubating sections with the secondary antibody alone, which yielded no observable staining or nonspecific fluorescence [71,72].

### 3.4. Procedure of Collagen Extraction from Fish Gastrointestinal Organs for Macromolecular Characterisation

Collagen extraction was performed following the method described by Abbas et al. (2022) [11]. Briefly, tissues from the oesophagus, stomach, and intestine were cut into small pieces; treated with 0.1 M NaOH to remove non-collagenous proteins; and defatted overnight using 10% butyl alcohol. Pepsin-soluble collagen (PSC) was then extracted by incubating the deproteinised and defatted tissues in 0.5 M acetic acid containing 0.1% (*w*/*v*) pepsin. After extraction, the supernatant was subjected to salting out using NaCl to a final concentration of 2.5 M in 0.05 M Tris buffer (pH 7.0). The precipitated collagen was dissolved in 0.5 M acetic acid, followed by buffer exchange using Amicon Ultra 0.5 mL centrifugal filters, 3 kDa MWCO (Merck KGaA, Darmstadt, Germany), and distilled water.

### 3.5. SDS-PAGE and Western Blot Procedure

SDS-PAGE was performed using an 8% resolving gel and a 6% stacking gel. After electrophoresis, the gel was stained overnight with 0.25% Coomassie Brilliant Blue R-250 solution and then destained with 5% ethanol in water until the protein bands were clearly visible [11].

For Western blotting, proteins were transferred onto a PVDF membrane using the wet transfer method. The membrane was blocked with 5% nonfat milk in PBS-T buffer for one hour and incubated overnight at 4 °C with the primary antibody diluted 1:1000 (Table 4). After washing three times for 10 min with PBS-T (137 mM NaCl, 2.7 mM KCl, 10 mM Na_2_HPO_4_, 0.05% Tween-20), the membrane was incubated with an HRP-conjugated secondary antibody for two hours at room temperature (Table 4). Following three additional washes, protein bands were visualised using an ECL substrate (Santa Cruz Biotechnology, #SC-2048, Dallas, TX, USA,) with a 1 min substrate incubation. Signal detection was subsequently carried out by exposing the membrane to X-ray film (Fuji, #RX1824) for exactly 1 min, and the film was developed with a Fuji Medical Film Processor FPM-100A (Fuji Photo Film Co., Tokyo, Japan). Housekeeping protein normalisation was not performed because the extraction protocol was optimised for collagen isolation rather than total cellular protein recovery. Therefore, Western blot analysis was used to confirm the presence of collagen in the digestive tissue [73]. Western blot bands were analysed using ImageJ software (v1.54) (NIH, Bethesda, MD, USA). Band intensities were quantified by measuring integrated density after the manual selection of regions of interest and background subtraction. The quantified values are expressed as net integrated density in arbitrary units (A.U.) to reflect the absolute band intensities of the α-chain and β-chain across the analysed tissues.

### 3.6. Collagen Quantification—Hydroxyproline Assay Procedure

Hydroxyproline content in the three digestive system regions (the oesophagus, stomach and intestine) was determined to represent the total amount of collagen. It is worth noting that this assay was applied directly to whole tissue samples following complete acid hydrolysis to determine the total theoretical collagen content present in the organs, rather than evaluating the efficiency of a physical extraction or isolation protocol. Briefly, 10 mg of tissue samples was weighed accurately, sealed in ampoule bottles, and hydrolysed in 100 µL of 37% HCl at 120 °C for 3 h. The cooled digestive supernatant was then processed according to the instructions of the hydroxyproline kit (catalogue number MAK008, Sigma-Aldrich, Merck, Darmstadt, Germany). A total of 15 individual fish specimens (n = 15 per organ) were used as distinct biological replicates to account for natural population variability. From each biological specimen, samples were processed and analysed in technical duplicates. The absorbance of the prepared samples was measured at 560 nm (A560).

### 3.7. Statistical Analysis

For the quantitative analysis of type I collagen expression in the digestive system of the greater weever, ImageJ software (National Institutes of Health, Bethesda, MD, USA) was used. To quantify the immunoreactive area of type I collagen, a total of 100 non-overlapping representative visual fields per organ were examined. Images (8-bit greyscale, 1608 × 1608 pixels) were captured with identical exposure times at an objective magnification of 40× and a wavelength of 488 nm. ImageJ software detected the percentage of type I collagen immunoreactive areas and fluorescence intensity in three regions (the oesophagus, stomach and intestine) within the digestive system. For immunoreactivity analysis, photomicrographs were first processed with a 5-pixel median filter to reduce noise and subsequently subjected to a standardised empirical thresholding procedure using the Triangle method algorithm with a fixed lower threshold cutoff set at 27 and the upper limit at 255 to measure the total percentage area covered by a positive signal [70,72,74]. This specific optimised threshold was determined through preliminary optimisations to ensure the complete exclusion of background autofluorescence while fully capturing the true positive collagen signal. To ensure objectivity and comparability across all groups, these exact thresholding and particle analysis parameters (size range: 0–infinity) were kept strictly uniform and applied systematically to every analysed image within a unified batch process, without any manual adjustments between the sessions. For the statistical analysis of the type I collagen area percentage, a one-way ANOVA followed by Tukey’s multiple comparison test was used to examine differences in expression signals among the oesophagus, stomach, and intestine. Additionally, a two-way ANOVA followed by Šídák’s multiple comparison test was performed to evaluate the amount of type I collagen in the gastrointestinal system, as determined by the hydroxyproline assay. Data were presented as the mean ± standard deviation (M ± SD). Statistical significance was considered at *p* < 0.05. Analysis was performed in GraphPad Prism 10.1.1 (GraphPad Software, Inc., San Diego, CA, USA).

## 4. Conclusions

This study provides the first characterisation and quantitative analysis of type I collagen in the digestive system of the greater weever (*T. draco*). Our findings delineate the qualitative and quantitative expression of type I collagen within the walls of the oesophagus, stomach, and intestine, defining these tissue-specific structural characteristics for the first time and establishing a reliable physiological framework for future research on collagen distribution in other tissues of this species. Furthermore, by focusing on commonly overlooked fishery waste, this work provides a valuable foundational framework for assessing the molecular potential of type I collagen from gastrointestinal by-products. While further research with expanded sample sizes and diversified extraction techniques is warranted to optimise isolated collagen yields for potential industrial scaling, our results strongly highlight the valorisation potential of these materials across various industries, fully supporting sustainable marine circular economy principles. Integrated with the recent discovery of bioactive peptides from the myofibrillar proteins of *T. draco*, our findings suggest that this species could represent a significant and sustainable source of biologically active molecules for future biomedical and industrial applications.

## Figures and Tables

**Table 1:** A quantitative analysis of type I collagen expression (% fluorescent area) in the digestive system of the greater weever (*T. draco*).

Parameter	Oesophagus	Stomach	Intestine	Statistical Test (*p*-Value)
N (Measurements)	100	100	100	—
Mean (% area) ± SD	1.069 ± 1.046	1.090 ± 1.053	0.932 ± 1.080	0.1315 (ns) ^1^
Std. Error (SEM)	0.1046	0.1053	0.1080	—
Normal distribution	Passed	Passed	Passed	>0.05 (ns) ^2^
Equality of Variances	—	—	—	0.0051 (**) ^3^

N: Total number of technical measurements performed across 15 biological replicates. SD—standard deviation ^1^. SEM: standard error of the mean; one-way ANOVA: (F(2, 27) = 2.189) ^2^. Shapiro–Wilk & D’Agostino-Pearson tests: Confirmed normal distribution for all groups ^3^. Brown–Forsythe test: Indicates significant heteroscedasticity (unequal variances) among the groups. ns—not statistically significant (*p* > 0.05). **: Statistically significant difference in variances (*p* < 0.01).

**Table 2:** Collagen content (*w*/*w* %) and hydroxyproline content (µg/mg) isolated from fish gastrointestinal organs. The values of both collagen and hydroxyproline (µg/mg) in the samples were calculated per milligram of the initial mass (10 mg). The average values (±SD) are the means of two replicates.

Gastrointestinal Organ	% Collagen	% Hydroxyproline
Oesophagus	3.396 ± 2.848	0.4551 ± 0.3816
Stomach	4.199 ± 3.537	0.5629 ± 0.4741
Intestine	1.713 ± 1.199	0.2297 ± 0.1605

**Table 3:** A two-way repeated measures ANOVA and Šídák’s post hoc multiple comparisons of hydroxyproline and type I collagen levels across the digestive tract organs of the greater weever (*T. draco*).

Statistical Analysis	Source of Variation/Comparison	DF	F-Value	*p*-Value	Significance
Two-way RM ANOVA	Column Factor (Organs)	2, 36	3.841	0.0308	*
	Row Factor (Analysis Type)	1, 18	18.55	0.0004	***
	Interaction (Organs × Type)	2, 36	2.240	0.1211	ns
	Subject (Paired replicates)	18, 36	2.167	0.0238	*
Post hoc Test (Šídák’s)	Oesophagus vs. Stomach	—	t = 0.877	0.7687	ns
(Organ Comparisons)	Oesophagus vs. Intestine	—	t = 1.838	0.2067	ns
	Stomach vs. Intestine	—	t = 2.716	0.0300	*

DF = degrees of freedom; F-distribution ratio; t: Student’s *t*-test statistic from Šídák’s post hoc test; ns = not significant, *p* > 0.05; * = *p* < 0.05; *** = *p* < 0.001; (N) = 15. The analysis is based on an experimental matrix of 19 paired biochemical datasets derived from the tissue samples (DF = 18 for the Subject factor, calculated as n − 1), encompassing 10 replicated measurements per analysis type for each examined organ.

**Table 4:** Primary and secondary antibodies.

	Antibody	Cat. Number	Host	Dilution	Produced by
primary	Anti-Fish Collagen Type I	CO20171-T-0.1	rabbit	1:80 1:1000	Nordic-MUbio, Susteren, The Netherlands
secondary	Alexa Fluor^®^ 488-conjugated AffiniPure Donkey Anti-Rabbit IgG (H+L)	711-545-152	donkey	1:300	Jackson Immuno Research Laboratories, Inc., Baltimore, PA, USA
secondary	Peroxidase AffiniPure^®^ Goat Anti-Rabbit IgG (H+L)	111-035-144	goat	1:10,000	Jackson Immuno Research Laboratories, Inc., Baltimore, PA, USA

## Data Availability

The original contributions presented in this study are included in the article. Further inquiries can be directed to the corresponding author.
